# TRial to Assess Implementation of New research in a primary care Setting (TRAINS): study protocol for a pragmatic cluster randomised controlled trial of an educational intervention to promote asthma prescription uptake in general practitioner practices

**DOI:** 10.1186/s13063-022-06864-y

**Published:** 2022-11-17

**Authors:** Rami A. Alyami, Rebecca Simpson, Phillip Oliver, Steven A. Julious

**Affiliations:** 1 School of Health and Related Research (ScHARR), University of Sheffield, Regent Court, 30 Regent Street, Sheffield, S1 4DA UK; 2 Respiratory Therapy Department, Faculty of Applied Medical Sciences, Jazan University, Jazan, Saudi Arabia; 3 Academic Unit of Primary Medical Care, Samuel Fox House Northern General Hospital, Herries Road, Sheffield, S5 7AU UK

**Keywords:** Asthma, Child, Childhood asthma, Asthma management, Cluster randomised controlled trial, GP practices, Public health intervention, Knowledge Translation, Knowledge Mobilisation, Implementation, Prescription pattern, Unscheduled medical contacts

## Abstract

**Background:**

There is a marked increase in unscheduled care visits in school-aged children with asthma after returning to school in September. This is potentially associated with children not taking their asthma preventer medication during the school summer holidays. A cluster randomised controlled trial (PLEASANT) was undertaken with 1279 school-age children in 141 general practices (71 on intervention and 70 on control) in England and Wales. It found that a simple letter sent from the family doctor during the school holidays to a parent with a child with asthma, informing them of the importance of taking asthma preventer medication during the summer relatively increased prescriptions by 30% in August and reduced medical contacts in the period September to December. Also, it is estimated there was a cost-saving of £36.07 per patient over the year. We aim to conduct a randomised trial to assess if informing GP practices of an evidence-based intervention improves the implementation of that intervention.

**Methods/design:**

The TRAINS study—TRial to Assess Implementation of New research in a primary care Setting—is a pragmatic cluster randomised implementation trial using routine data. A total of 1389 general practitioner (GP) practices in England will be included into the trial; 694 GP practices will be randomised to the intervention group and 695 control group of usual care. The Clinical Practice Research Datalink (CPRD) will send the intervention and obtain all data for the study, including prescription and primary care contacts data. The intervention will be sent in June 2021 by postal and email to the asthma lead and/or practice manager. The intervention is a letter to GPs informing them of the PLEASANT study findings with recommendations. It will come with an information leaflet about PLEASANT and a suggested reminder letter and SMS text template.

**Discussion:**

The trial will assess if informing GP practices of the PLEASANT trial results will increase prescription uptake before the start of the school year. The hope is that the intervention will increase the implementation of PLEASANT work and then increase prescription uptake during the summer holiday prior to the start of school.

**Trial registration:**

ClinicalTrials.gov ID: NCT05226091

**Supplementary Information:**

The online version contains supplementary material available at 10.1186/s13063-022-06864-y.

## Background

Asthma is a chronic respiratory disease that affects adults and children. It is a disease of the airways that results in inflammation and narrowing of airways associated with wheezing, coughing and shortness of breath [1]. According to Asthma UK, approximately 5.4 million people in the UK have asthma, 4.3 million adults [1 in 12] and 1.1 million children [1 in 11] [2]. Asthma is the most common chronic disorder that affects children worldwide [3]. The National Review of Asthma Deaths (NRAD) reported that 45% of people who died from asthma between 2012 and 2013 could have been prevented [4]. Worldwide, children in the UK suffer from the highest prevalence of asthma symptoms. Every 20 min, a child in the UK is hospitalised from an asthma attack, and 185 Adults and children are admitted every day [5].

People with asthma should be assessed, and their medications should undergo a continuous assessment to manage their disease and reduce the disease burden [6]. In the UK, the National Institute of Health and Care Excellence (NICE) recommends that patients with asthma should be followed up at least once a year to determine whether their treatment needs to be modified [7]. According to current clinical guidelines by the British Thoracic Society (BTS) and NICE, all individuals who have been hospitalised with an asthma attack should receive a follow-up assessment in primary care within two working days of discharge to evaluate asthma control and ensure symptoms are resolved. Despite this, 64% of respondents to the 2018 National Asthma Survey did not follow-up with their GP after an attack, and many patients were unaware this was required [7, 8].

**Fig. 1:**
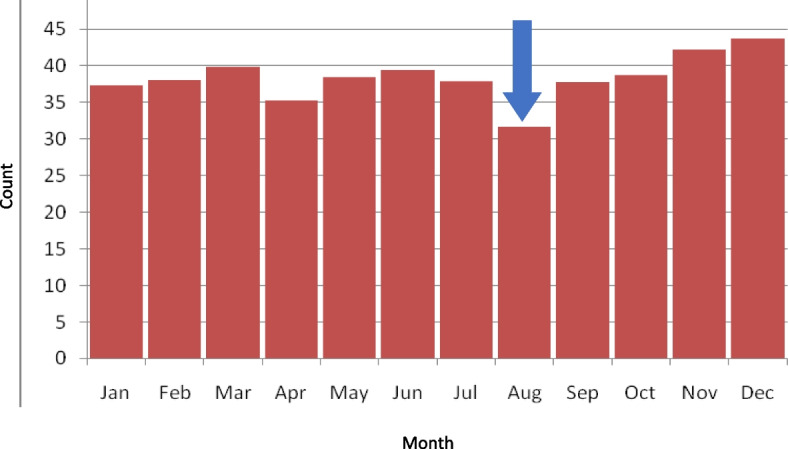
Number of asthma preventer prescription uptake by month in England [11]

Non-adherence to asthma medication or inhaler mishandling increases morbidity, mortality, and hospital admission [9]. Previous work by our research group has shown that school-aged children with asthma are twice as likely to have an unscheduled care contact after returning to school in September as children without. Furthermore, 30% will have an unplanned medical visit in the month of September [10]. The increase in unscheduled contacts is associated with a drop in the number of asthma-related prescriptions for preventer medications during the preceding summer holidays (Fig. 1) [11].

This research provided the rationale for the PLEASANT study (*P*reventing and *L*essening *E*xacerbations of *A*sthma in *S*chool-age children *A*ssociated with a *N*ew *T*erm) [12]. PLEASANT was a cluster randomised trial to assess whether a letter sent from a GP practice at the start of the summer vacation reminding parents of children with asthma of the necessity of continuing to take their medication during the summer holidays. The study evaluated whether the letter increased prescriptions in August and reduced unscheduled contacts after returning to school in September [13].

**Fig. 2:**
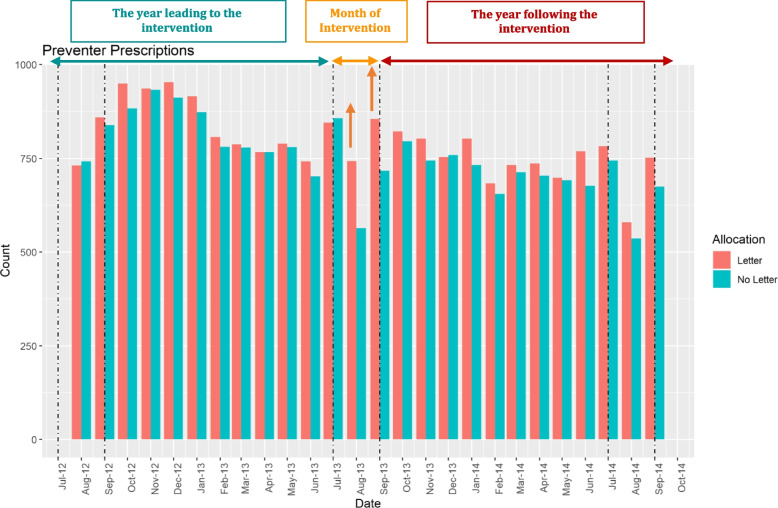
Prescriptions uptake over two years [14]

**Fig. 3:**
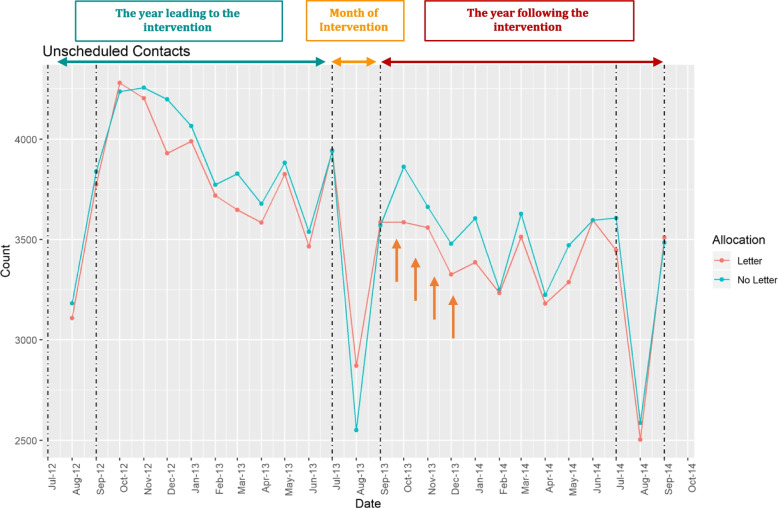
Unscheduled contacts over two-years [14]

The PLEASANT study included 1279 school-age children from 141 general practices (71 on intervention and 70 on control) in England and Wales. Accordingly, the results of PLEASANT showed that the letter relatively increased prescription uptake in August by 30%—an absolute increase of 4% (Fig. 2)—and reduced the unscheduled medical contacts after the return to school the period September to December (Fig. 3). Although the effects were small the intervention was cost-saving—saving £36.07 per patient over 1 year [14].

The PLEASANT trial showed that a simple, cost-effective intervention could increase preventer prescriptions and reduce unscheduled primary care contacts. There is, thus, a clear clinical benefit in translating the results of this clinical research from publication to practice. This leads to the motivation for the TRAINS project.

**Fig. 4:**
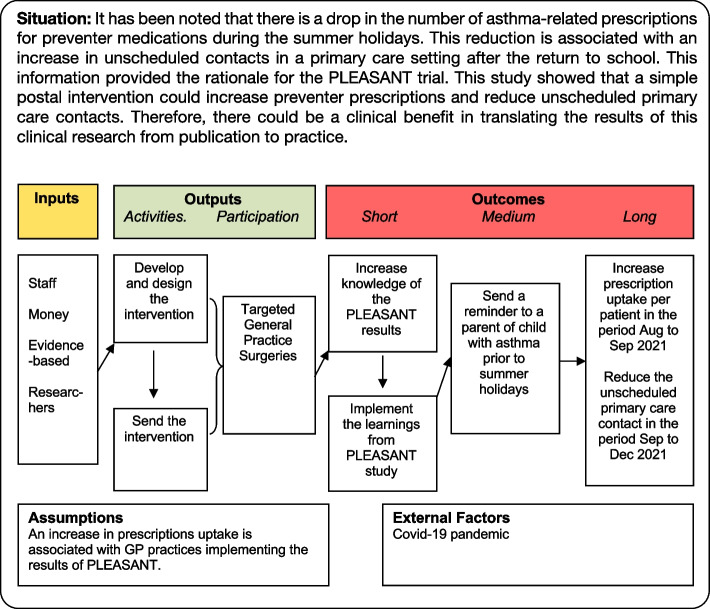
A logic model for the TRAINS trial

The TRAINS trial will assess the effect of an intervention sent to GP practices (Fig. 4). The intervention will provide information on the PLEASANT trial results and be sent by email and mail to the asthma lead and/or practice manager.

### Primary research aim

The aim of this trial is to assess if informing GP practices about the findings of PLEASANT increases prescription uptake in school-age children with asthma. The primary objective of the trial is to determine whether the intervention increases asthma prescription uptake in children with asthma in August and September.

### Research question

This project will address the following research question:

“Does Informing General Practitioners about the Results of PLEASANT Trial Prevent Summer Holiday the Drop in Prescription Uptake by the Parents/Carers of School-Age Children with Asthma?”

### Rationale

The PLEASANT trial showed that a simple postal intervention effectively improves asthma outcomes in children in primary care. The objective of the current study is to conduct a randomised trial to assess if informing GP practices of an evidence-based intervention results in the implementation of that intervention.

## Methods and analysis

### Trial design

**Fig. 5:**
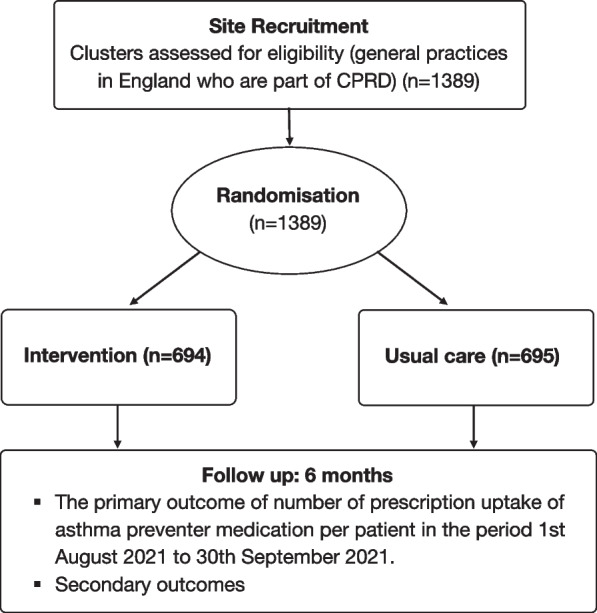
Trial summary

TRAINS will be a cluster randomised controlled parallel-group trial using routinely collected data. There are approximately 1389 general practices contribute to CPRD Aurum database in England. Six hundred ninety-four GPs will be randomised to the intervention group and 695 to the control group of usual care (Fig. 5). Data will be extracted over a 6 months period after the implementation. Cluster randomisation was chosen as the intervention will be delivered at the level of the practice.

### Designing the intervention

**Fig. 6:**
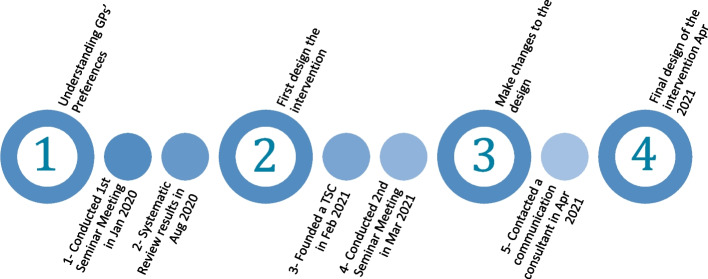
Flowchart of the development of the intervention over time

The intervention (letter) has been designed through four phases based on five steps: an initial meeting with GPs, systematic review, trial steering committee, follow-up GP meeting, and a communication consultant (Fig. 6).

In the first phase of designing the intervention, we conducted a seminar meeting with the Academic Unit of Primary Care in Sheffield to understand what design and content attributes are perceived by GPs. Then, we conducted a systematic review to ensure the study had not been done and to help develop the intervention. Based on the seminar recommendations and the systematic review results, two draft intervention templates were designed in September 2020. A long version and a short version. The long version included graphs from the PLEASANT study results alongside detailed text and the original reminder letter that was used in the PLEASANT trial. The short version included text but did not include graphs and links to download the PLEASANT’s reminder letter.

In the second phase of the design, we founded a trial steering committee and carried out a second seminar meeting to get feedback on the first two templates (long and short). Following this, recommendations were as follows: to send the short version of the intervention, to target the asthma clinical lead and practice manager, and to include a reminder letter and SMS text template to simplify and increase the possibility of the implementation. In addition, it was advised to attach a leaflet about PLEASANT Trial to support the letter and provide a link to the PLEASANT trial website for further details on PLEASANT and its publications and documents.

In the third phase of design, we contacted a communication and copywriting consultant, who has written extensively for both NHS England and Leicester City’s Public and Patient magazine, to enhance the language and layout of the intervention letter [15]. The short letter with a leaflet and suggested reminders (letter and SMS text for GPs to use) was shown to the communication consultant. The consultant edited the material to provide punchy and persuasive messaging for a series of communication interventions by letter, email, and leaflet. In addition, guidance was delivered on a high-impact visual layout and style of the letters and leaflet.

In the fourth phase of the design, the final version of the intervention was designed in April 2021. The intervention consists of a letter to GPs with recommendations and links, a supported leaflet about the PLEASANT study for more information about the findings, reminder templates for GPs to use and make it easier for implementation, and an email to GPs with the intervention materials. The final version of the intervention can be found in the supplemental material (Additional file 1).

### The intervention

Selected GP practices currently registered with CPRD and randomised to the intervention arm will receive correspondence twice, once by email and once by mail. The intervention is a letter that highlights the drop in prescription uptake for children with asthma during the summer holidays and introduces the results of the PLEASANT trial. In addition, it advises GP practices on the effect of reminding parents of children with asthma to continue taking their asthma preventer medications during the summer holidays. It will be sent with the assistance of the CPRD to the CPRD contact lead at GPs along with the named to the practice manager and asthma lead. The plan, subject to confirmation and amendment, is that the intervention will be sent by July 2021, which is the advice from the trial steering committee (TSC). Given the timing of the intervention, the hope is that GP practices will then send their correspondence to parents during the school holidays. It is hoped the intervention can be timed to enable sufficient time for GP practices to implement the advice from the intervention.

As this is an implementation study, the correspondence to GP practices is advisory, and the decision on implementation is down to the individual GP practices.

### Primary outcome measure

- The proportion of children with asthma who have a prescription for an asthma preventer medication from 1 August 2021 to 30 September 2021.

### Secondary outcome measures

1. The number of asthma preventer medication prescriptions per school-aged child with asthma patient from 1 August 2021 to 30 September 2021
2. The number of prescription uptake of asthma preventer medication per patient in the month of August 2021
3. The number of prescription uptake of asthma preventer medication per patient in the month of September 2021
4. The proportion of children who have a prescription for asthma preventer medication per patient in the month of August 2021
5. The proportion of children who have a prescription for asthma preventer medication per patient in the month of September 2021
6. The number of prescription uptake of asthma preventer medication in the 6 months following the intervention 1 July 2021
7. The proportion of patients with unscheduled medical contact from 1 September 2021 to 31 December 2021 and the individual months of 1 September 2021 to 31 December 2021
8. The number of unscheduled medical contact per patient from 1 September 2021 to 31 December 2021 and the individual months from 1 September 2021 to 31 December 2021
9. The proportion of patients with a medical contact (either unscheduled or scheduled) from 1 September 2021 to 31 December 2021 and the individual months from 1 September 2021 to 31 December 2021
10. The total number of medical contact (either unscheduled or scheduled) per patient in the period 1 September 2021 to 31 December 2021 and the individual months of 1 September 2021 to 31 December 2021
11. The proportion of patients who have an unscheduled medical contact in the period 1 September 2021 to 31 December 2021 and the individual months of 1 September 2021 to 31 December 2021 associated with a respiratory diagnosis
12. The number of unscheduled medical contacts per patient and from 1 September 2021 to 31 December 2021 and the individual months of 1 September 2021 to 31 December 2021 associated with a respiratory diagnosis

### Setting and site recruitment

The study will be conducted within GP practices in England that are currently contributing to CPRD Aurum database. The GP practices will be randomised to receive the intervention, and they will not be aware that they are in the study. Approximately 1389 sites are expected to be involved in this trial based on current active CPRD GP practice sites.

### Randomisation and allocation concealment

Practices will be allocated 1:1 to intervention or control to ensure an equivalent sample size in each arm of the study in terms of the number of children with asthma. The randomisation will be stratified by the size of the practice as assessed by decile within the CPRD. All the practices in the study will be known from the CPRD and will not be prospectively recruited. GP practices randomised to the intervention will receive a postal bulletin and an email advising them to implement the study. GP practices randomised to control will not receive postal bulletin or email, and they will continue with usual care.

For practices included in the study, randomisation will be undertaken by study statistician using bespoke Excel spreadsheet. A file with anonymous practice identification codes and the stratification number will be provided to the CPRD. The statistician will not receive any information that would identify the practices.

### Population

The target population for the intervention will be general practices who are currently contributing to CPRD in England.

### Practices inclusion criteria

General practices who are currently contributing to CPRD Aurum database in England on or before the intervention were included.

### Practice exclusion criteria

General practices that are not in England, practices that leave CPRD after the intervention and before the end of follow-up, and practices that merge after the intervention and before the end of follow-up (where the merging practices were in different study arms) were not included.

### Inclusion criteria for the data extraction from CPRD

School-aged children with asthma aged between 4 and 16 years old as of 1 September 2021 with a coded diagnosis of asthma who have been prescribed asthma medication in the last 12 months were included.

### Data source

The data for this study will be routinely collected data extracted from the Clinical Practice Research Datalink—Aurum (CPRD Aurum) database. This database covers approximately 20% of the UK’s population, with over 1300 general practices participating [16]. CPRD Aurum is a database of de-identified patient data that can capture all medical contacts, from prescription requests to out-of-hours contacts, along with the reason for the contact. The data is contributed by general practices.

CPRD will extract data from primary care medical 6 months post-follow-up. The research team will receive all data fully anonymised and will not have access to any patient identifiable data.

### Allocation of preventer prescription

All medicines that have been prescribed will be reviewed, and identify all the asthma medications that have been prescribed. The asthma preventer prescription will be used to determine the primary outcome, whilst prescriptions for short-course oral steroids will be used to inform the definition of the secondary outcomes, including unscheduled care.

### Allocation of scheduled vs. unscheduled contacts

To ascertain whether a clinical contact was scheduled or unscheduled, we will use the contact codes used by the GP practice captured within the practice database. We are defining a scheduled contact as any contact that is part of the patient’s planned care, such as an asthma review, a medical review, repeat prescription, or immunisation. An unscheduled contact will be any contact not part of their care plan that is either patient initiated or as a result of illness. The decision (scheduled vs unscheduled) will be assisted by any prescription issues associated with the contact. For example, if there is a prescription for a short course of oral corticosteroids, this would infer both the contact is unscheduled, and it had a respiratory diagnosis. To ensure the allocation of scheduled and unscheduled contacts, we will draw on our experience of the coding for the PLEASANT study. Also, as part of the study team, we have a current GP to consult at each stage. In addition, the trial steering committee comprises two GPs and a practice nurse who also could advise us.

### Covariates

We plan to include practice characteristics including the practice size and the deprivation score associated with the practice. The covariates to be included in the statistical model will consist of the age, sex, and ethnic group of the child. The baseline for the primary outcome of prescriptions, the number of asthma preventer prescriptions, will be the years 2019 and 2020 in August and September. To account for the effect of practice clustering, GP will be included as a random effect. The same covariates will be collected for both intervention and non-intervention arms in the study.

### Statistics

#### Sample size

The sample size is based on feasibility and the anticipated number of practices providing data to the CPRD. We anticipate a sample size of approximately 1389 GP practices (694 on intervention and 695 on control). Alongside this, from the previous study (PLEASANT), we also anticipate 85 school-age children having asthma per practice [14]. Assuming an expected rate of 30% of people collecting their prescription and an intraclass correlation of 0.03, it is anticipated that the precision of the estimates in the study—estimated as a half-width of a 95% confidence interval—will be 1%.

#### Data analysis

Statistical methods appropriate to a cluster randomised trial will be used for the analysis of the data. The statistical analysis will be performed on an intention-to-treat-basis and reported according to CONSORT guidelines for Cluster RCTs [17]. A dropout of practices is not expected. Some practices may merge, close, or split. Small numbers are likely to be involved, but no special treatment is necessary and no imputation is planned.

#### Primary analysis

The proportion of children having a preventer prescription collected within the primary time period per child will be analysed using a random effect logistic regression model with the individual’s age, sex, number of preventer prescriptions in the baseline period as covariates, the trial arm (intervention or control) as a fixed effect, and the design/cluster effect of general practice as a random effect. Effect sizes (assessed as an odds-ratio) and 95% confidence intervals for the primary endpoint will be presented. The number of prescriptions per child will be similarly analysed separately for each time period using negative binomial regression.

#### Secondary analysis

The secondary endpoints—the number of unscheduled contacts per child and the proportion of patients with unscheduled medical contact in the different time intervals—will be similarly analysed to the primary endpoint.

#### Subgroup analyses

Subgroup analyses will be performed for the primary outcomes. The subgroups are defined as follows:

- Patient-level characteristics:
  - Age (< 5, 5–11, and 11+)
  - Sex (boys vs. girls)
  - Ethnic group
  - Asthma diagnosis (< 5, 5+)
- Read receipt vs not read (email that sent to GPs)
- GP IMD quintile

Each subgroup analysis will be carried out by adding the subgroup variable into the main logistic regression model. Summary measures for each group will comprise prescription uptake counts and percentages within each arm, as well as the OR for effect with a 95% CI.

#### Information governance

TRAINS (TSC) was established to oversee the study from an independent perspective. The TSC consist of two academic GP, practice nurse, practice manager, and statistician. The role of the TSC is to provide advice on the study, ensure the delivery of the intervention, and achieve the project outcomes. Also, it focused on the intervention design. The TRAINS Trial Steering Committee provides guidance, support, and input to the development of the trial.

#### Patient and public involvement in the design of the trial

During the design of the TRAINS trial, two seminar meetings were arranged with the Academic Unit of Primary Care. The first was held in January 2020, and the second seminar was planned for March 2021, involving 16 GPs plus researchers and teaching staff. These events were used to investigate the designing of the intervention, to get feedback on to whom the letter should be addressed (i.e. addressed to practice manager, asthma lead, or both), to discuss the timing of sending the intervention (i.e. June and July). The first initial PPI event was written up and uploaded onto the figshare website [18]*.*

## Discussion

TRAINS is a large pragmatic cluster randomised controlled trial that will assess the effectiveness of a brief postal intervention sent to GP practices informing them about the PLEASANT trial results, evidenced by an increase in prescription uptake during the summer holiday. The advantage of the study design is that it has an efficient study design using the CPRD to get the data. The GP practices will not know if they are in the study and will not be recruited into the trial for data collection. GP practices are free to implement the learnings from PLEASANT as they see fit.

The strengths of the study that as CPRD covers approximately 20% of the UK’s population, the study will have a large sample size and generalisable data [16]. This will allow for several relevant subgroup analyses to be performed with sufficient statistical power.

Limitations of the study include the COVID-19 pandemic effect, which has impacted the design of the intervention. We would have liked to undertake more seminars and meet more primary care doctors, nurses, and practice managers to enhance and improve the intervention and to have done it face to face. This has not proved to be not possible due to the lockdown to contain the epidemic, which did not let me advance the intervention as we planned and wanted. Also, we had to add an extra baseline to account as 2020 was not a standard year.

Another limitation of the study is that we have no plans to contact the practices to assess if the intervention was sent. The assumption will be an increase in prescriptions is due to practices sending the intervention.

### Ethical review

Ethical approval for this trial was obtained from the University of Sheffield Research Ethics Committee (Reference Number\ 037412) (Approved 26 April 2021).

Ethical approval has been obtained through the Independent Scientific Advisory Committee (ISAC) for Clinical Practice Research Datalink (CPRD) database research (Protocol reference Id\ 21_000436) (Approved 16 June 2021).

### Protocol amendments

A protocol amendment will be required if modifications might affect the conduct, benefit of the study or patient safety, including changes in the study objectives, study design, patient population, sample size, study procedures, or significant administrative aspects. This amendment will be agreed by the trial team and approved by the University of Sheffield Research Ethics Committee and the Independent Scientific Advisory Committee (ISAC) for Clinical Practice Research Datalink (CPRD) database. A protocol administrative change involves minor corrections or clarifications that do not affect the research process. These changes will be agreed upon by trial team and documented and may be notified to the Ethics Committees.

#### Trial status

The trial is now at the stage where we have received the routine data, and it is being processed and cleaned before analysis can begin.

## Supplementary Information

**Additional file 1.** Letter to GPs.

## Data Availability

In this study, data sharing can be obtained by contacting CPRD as a third party.

## Abbreviations

GPs: General practitioners
GP: General practice (GP surgery)
ICC: Intraclass correlation
PPI: Patient and public involvement
CPRD: Clinical practice research datalink
PLEASANT: Preventing and Lessening Exacerbations of Asthma in School-age children Associated with a New Term
TRAINS: TRial to Assess Implementation of New research in a primary care Setting
CONSORT: Consolidated Standards of Reporting Trials
TSC: Trail Steering Committee

## Glossary

Asthma: Asthma refers to a chronic inflammatory illness of the airways. Chronic inflammation is linked with airway hyper-responsiveness, resulting in recurrent episodes of symptoms such as tightness in the chest, breathlessness, wheezing, and coughing with different intensities over time [19].
Asthma preventer medication: A type of asthma treatment that is used to prevent and control asthmatic symptoms should be taken daily. In this trial, we are looking for when a child collects their or her asthma preventer medication during, and just after, the summer holidays (1 August 2021–30 September 2021).
Scheduled medical contact: A planned appointment for a child with asthma with the GP practice as part of their asthma care plan.
Unscheduled medical contact: An unplanned visit by a child with asthma to a GP practice or other health setting (e.g. hospital contacts, walk-in clinics)
Unscheduled medical contact: with a respiratory diagnosis: An unplanned visit by a child with asthma to a GP practice or other health setting (e.g. hospital contacts, walk-in clinics) for which there is a respiratory diagnosis
