# Supplementary material for: TRial to Assess Implementation of New research in a primary care Setting (TRAINS): study protocol for a pragmatic cluster randomised controlled trial of an educational intervention to promote asthma prescription uptake in general practitioner practices
Source: Trials. 2022 Nov 17;23:947. doi: 10.1186/s13063-022-06864-y (PMC9670052; doi:10.1186/s13063-022-06864-y)
Supplement: Supplementary file 1 — Additional file 1. Letter to GPs. [file 13063_2022_6864_MOESM1_ESM.pdf]

Letter to GPs

The University Of Sheffield.

The University of Sheffield
School of Health and
Related Research
Regent Court
30 Regent St
Sheffield
S1 4DA

To The Practice Manager and Asthma Lead,

We can help you to tackle peaks in asthma and improve outcomes for your young patients this summer.

As the asthma lead for your practice, I am writing to you with recommendations that will help you and your GP practice to reduce serious, asthma-related exacerbations in the young patients with asthma that you support.

Did you know?

Summer holidays are key for young people with asthma

The summer holidays are a crucial time for children with asthma to take their preventer medication ahead of the return to school, yet it often gets forgotten. With your help, a simple reminder to the parents of your young patients with asthma can **significantly reduce the number of serious medical attendances, hospital admissions and the associated costs**. Our recent University of Sheffield study of young patients with asthma supports this, as you'll see below.

How can we help you?

In one simple step

We are recommending that asthma leads at GP surgeries across England send a reminder letter or SMS text **in July.** This is to remind the parents or guardians of all school-age children with asthma to take their asthma medication during the summer break.

We've attached a sample reminder letter and SMS text.

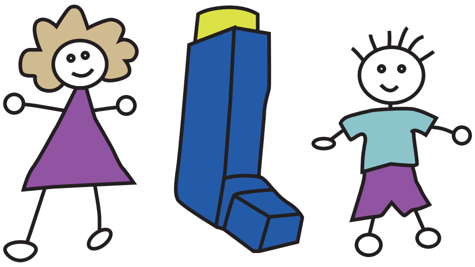

PLEASANT

What did our asthma study show?

Significant benefits of summer parent reminders

Initial findings of our asthma study showed a marked increase in unscheduled medical care for school-age children with asthma after they returned to school in September. It is thought that this is partially due to children not taking their asthma preventer medication during the summer holidays.

Our study found that a simple reminder letter sent by GPs to parents and guardians of children with asthma, reminding them to take their asthma medication during the summer holidays has resulted in:

A reduction in unscheduled medical contacts after the return to school in the period September to December.

Increased prescription uptake in August by 30%.

Cost savings of £36 per child.

Who can I contact about the asthma study?

If you would like more information about our asthma study or have any questions, please contact us at:
rahalyami1@sheffield.ac.uk

With one simple reminder message, you and your GP surgery can enable your young patients to manage their asthma better, ahead of the return to school.

To find out more about our asthma study known as **PLEASANT**, please read the attached leaflet or visit:
https://www.sheffield.ac.uk/scharr/research/centres/ctru/pleasant

Yours faithfully,

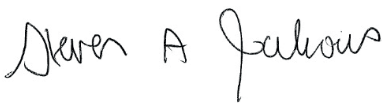

Professor Steven A. Julious
The University of Sheffield

Reference:

Julious, S.A., Horspool, M.J., Davis, S., Franklin, M., Smithson, W.H., Norman, P., Simpson, R.M., Elphick, H., Bortolami, O. and Cooper, C., 2018. Open-label, cluster randomised controlled trial and economic evaluation of a brief letter from a GP on unscheduled medical contacts associated with the start of the school year: the PLEASANT trial. BMJ open, 8(4).

Supported Leaflet

How can our findings help you and your patients with asthma?

In one simple step

We are recommending that asthma leads at GP surgeries across England send a reminder letter or SMS text **in July**. This is to remind the parents or guardians of all school-age children with asthma to take their asthma medication during the summer break. If you'

Click to download the letter or download the SMS text template.

Who can I contact about this asthma study?

If you would like more information or have any questions about our asthma study, please contact us at rahalyami1@sheffield.ac.uk

With one simple reminder message, you and your GP surgery can enable your young patients to manage their asthma better ahead of the return to school.

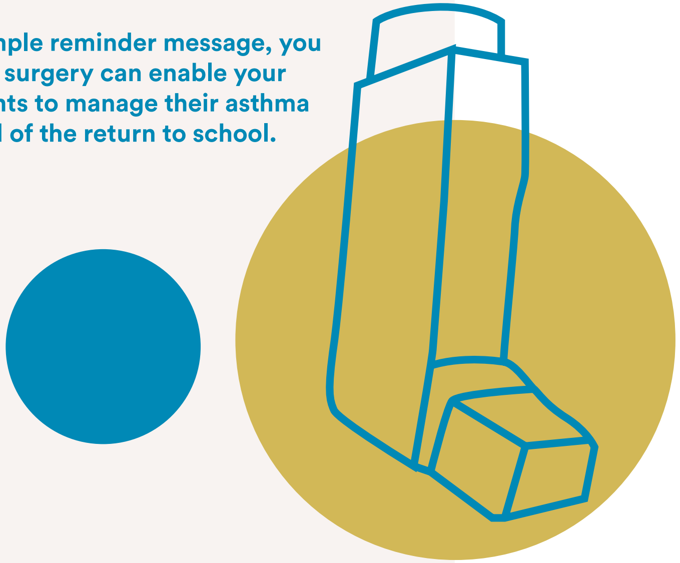

Publications

Julious SA, Horspool MJ, Davis S, Franklin M, Smithson WH, Norman P et al (2018). Open-label, cluster randomised controlled trial and economic evaluation of a brief letter from a GP on unscheduled medical contacts associated with the start of the school year: the PLEASANT trial. BMJ Open, 8(4), e017367.

Franklin M, Davis S, Horspool M, Kua WS & Julious S (2017). Economic Evaluations Alongside Efficient Study Designs Using Large Observational Datasets: the PLEASANT Trial Case Study. PharmacoEconomics, 35(5), 561-573.

Julious SA, Horspool MJ, Davis S, Bradburn M, Norman P, Shephard N, Cooper CL et al (2017) PLEASANT: Preventing and Lessening Exacerbations of Asthma in School-age children Associated with a New Term - a cluster randomised controlled trial and economic evaluation.. Health Technology Assessment, 20(93), 1-154.

Preventing and Lessening Exacerbations of Asthma in School-age children Associated with a New Term

Information for asthma leads and practice managers in GP surgeries.

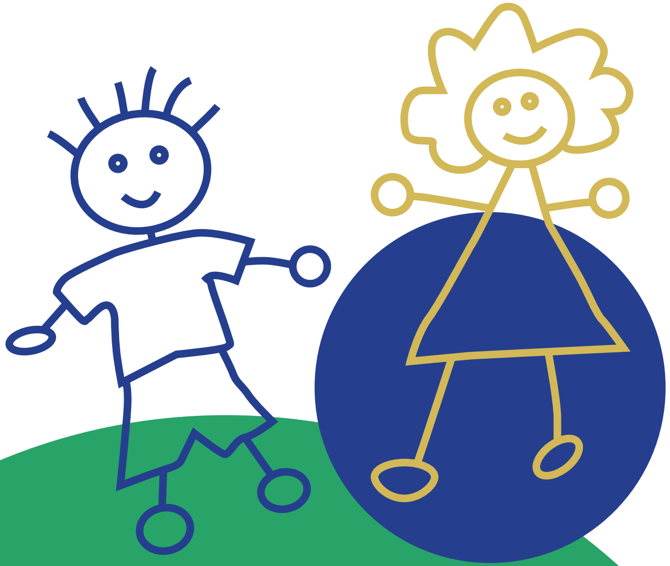

Many thanks for your interest in our University of Sheffield study of young patients with asthma.

If you're reading this leaflet, you've probably already received a letter from us highlighting how we can help you to reduce peaks in asthma for your young patients this summer.

This leaflet is for asthma leads like you across England, who'd like to know more about the study behind our summer awareness campaign for your young patients with asthma.

Our findings show that with your help, a simple reminder to the parents or guardians of your young patients with asthma can **reduce unscheduled care** when they return to school. Read on to find out more.

Asthma in young people – four findings to help you and them

The summer holidays are a crucial time for children with asthma to take their preventer medication ahead of the return to school, yet it often gets forgotten.

- **The return to school in September is a time when asthma gets worse for children** and there's an increase in medical help. This may be due to their contact with infections as they begin to mix with children in school at the start of the new school year.
- Our research shows **that children with asthma are twice as likely to see their doctor after the school return** compared to children without asthma.
- During the summer holidays, there's a significant drop in the number of prescriptions for asthma medication that are collected. **Children that aren't collecting their prescriptions are more likely to see their doctor.**
- **Communications which encourage school-age children to continue their prescribed medication, can make a real difference in helping them to manage their asthma.**

What did our asthma study show?

Significant benefits of summer parent reminders.

We carried out a trial of 12,179 school-age children in 141 GP practices across England and Wales, chosen at random.
Our study found that a simple reminder letter sent by the family GP to parents or guardians of children with asthma, asking them to make sure their child takes their preventer medication during the summer holidays, has resulted in:

A reduction in unscheduled medical contacts after the return to school in the period September to December.

Increased prescription uptake in August by 30%.

Cost savings of £36 per child.

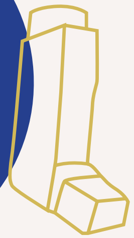

To find out more about our asthma study known as **PLEASANT**, please visit: https://www.sheffield.ac.uk/scharr/research/centres/ctru/pleasant

Email to GPs

Subject: This summer help manage asthma in your young patients

To the Practice Manager and Asthma Lead,

I am writing to you with recommendations that will help you and your GP practice to reduce serious, asthma-related exacerbations in your young patients with asthma, ahead of the return to school. (Please READ the attached letter)

With one simple reminder message this summer, you and your GP surgery can enable your young patients to manage their asthma better.

Here's why:

- The return to school in September is a time when asthma gets worse for children and there's an increase in medical help.
- During the summer holidays, there's a significant drop in the number of prescriptions for asthma medication that are collected.
- The summer holidays are a crucial time for children with asthma to take their preventer medication ahead of the return to school, yet often it gets forgotten.

How can we help you?

We are recommending that asthma leads like you at GP surgeries across England send a simple reminder letter or SMS text **in July.** This is to remind the parents or guardians of all school-age children with asthma to take their asthma medication during the summer break.

We've attached a sample reminder letter and SMS text (see attached). Alternatively click to download the letter or download the SMS text template.

With your help, this simple reminder to the parents of your young patients with asthma can **significantly reduce the number of serious medical attendances, hospital admissions and the associated costs.** Our recent University of Sheffield study (PLEASANT) of young patients with asthma supports this. You can find out more by reading the attached leaflet or visiting: https://www.sheffield.ac.uk/scharr/research/centres/ctru/pleasant

Who can you contact about our asthma study?

If you would like more information about our asthma study or have any questions, please contact us at: rahalyami1@sheffield.ac.uk

With one reminder, you and your GP surgery can make a difference in helping your young patients to manage their asthma better and have a safer return to school.

Yours faithfully,

Steven

Professor Steven A. Julious

The University of Sheffield

Suggested Reminder Letter

GP letterhead

< Address line 1>
< Address line 2>
< Address line 3>
< Address line 4>

<Insert Date>

Dear Parent

This summer holiday you can help prevent your child's asthma

Did you know that the summer holidays are a crucial time for continuing your child's asthma medication?

The return to school in September is a time when asthma often gets worse for young people. This may be due to contact with infections at the start of the new school year. But don't worry, act now and you can make the difference this summer.

How can I help prevent my child's asthma?

This summer, simply make sure you're child continues to take their asthma medication, as per their normal prescription.

What if my child has stopped taking their medication?

If your child has stopped taking their medication over the summer holidays, start it again as soon as possible. If they're short of medication, or you're not sure of the proper dose, please get in touch with us.

You can reduce the chances of your child getting poorly and help them have a safer return to school.

Yours sincerely

<Name of Doctor>

Suggested SMS Text Reminder

SMS Text (245 characters)

Reminder from your GP surgery: During the summer holidays, please make sure your child takes their asthma medication as per their usual prescription. You can help them have a safer return to school and reduce the chances of them getting poorly.
